# Mobile Phone-Based mHealth Approaches for Public Health Surveillance in Sub-Saharan Africa: A Systematic Review

**DOI:** 10.3390/ijerph111111559

**Published:** 2014-11-12

**Authors:** Johanna Brinkel, Alexander Krämer, Ralf Krumkamp, Jürgen May, Julius Fobil

**Affiliations:** 1 Department of Public Health Medicine, School of Public Health, University of Bielefeld, P.O. Box 100131, D-33501 Bielefeld, Germany; E-Mail: alexander.kraemer@uni-bielefeld.de; 2 Department of Biological, Environmental, Occupational Health Sciences, School of Public Health, University of Ghana, P.O. Box LG13, Legon, Ghana; E-Mail: jfobil@ug.edu.gh; 3 Infectious Disease Epidemiology Unit, Bernhard Nocht Institute for Tropical Medicine, Bernhard Nocht-Str. 74, D-20359 Hamburg, Germany; E-Mails: krumkamp@bnitm.de (R.K.); may@bnitm.de (J.M.)

**Keywords:** mobile health, eHealth, sub-Saharan Africa, mobile phone, cellular phone, surveillance, monitoring

## Abstract

Whereas mobile phone-based surveillance has the potential to provide real-time validated data for disease clustering and prompt respond and investigation, little evidence is available on current practice in sub-Sahara Africa. The objective of this review was to examine mobile phone-based mHealth interventions for Public Health surveillance in the region. We conducted electronic search in MEDLINE, EMBASE, IEE Xplore, African Index Medicus (AIM), BioMed Central, PubMed Central (PMC), the Public Library of Science (PLoS) and IRIS for publications used in the review. In all, a total of nine studies were included which focused on infectious disease surveillance of malaria (*n* = 3), tuberculosis (*n* = 1) and influenza-like illnesses (*n* = 1) as well as on non-infectious disease surveillance of child malnutrition (*n* = 2), maternal health (*n* = 1) and routine surveillance of various diseases and symptoms (*n* = 1). Our review revealed that mobile phone-based surveillance projects in the sub-Saharan African countries are on small scale, fragmented and not well documented. We conclude by advocating for a strong drive for more research in the applied field as well as a better reporting of lessons learned in order to create an epistemic community to help build a more evidence-based field of practice in mHealth surveillance in the region.

## 1. Background

The application of mobile and wireless technology in the health sector has the potential to change the face of global health systems [1,2]. That is reasoned by the powerful combination of four facts; (a) mobile technology has experienced a rapid technical development (b) falling market prices of the products, (c) increasing network coverage, and (d) an explosive increase of cell phones user rates all over the world [3]. Mobile health (mHealth) has a great potential to deliver life-saving information even in the most remote and resource poor settings in developing countries and can serve as an access point of national surveillance systems. Additionally, it offers an effective contribution to public health initiatives in support of achieving the health related Millennium Development Goals (MDGs) while being economical, effective and sustainable [1,3]. While no universal definition of mobile health has been established [1], it has been described as a sub-segment of electronic health (eHealth) [2] which provides medical and public health practice supported by mobile devices, such as mobile phones, smart phones, patient monitoring devices, Personal Digital Assistants (PDAs), as well as laptops and tablets PCs [4,5]. 

In spite of the demonstrated applicability to the health sector during the last decades, health systems in sub-Saharan African countries continue to face challenges in disease surveillance and infectious disease outbreak investigation [6]. Health surveillance in the region is then characterized by high costs which are directly linked to logistical, financial and infrastructural provisions with disease monitoring remaining a major public health challenge. In 1998 the African regional office of the World Health Organization (WHO) established the Integrated Disease Surveillance and Response (IDSR) regional framework for strengthening national public health surveillance capabilities at all levels in Africa [7]. IDSR provides the functions, activities, and skills required for implementing coordinated surveillance systems for each health level and implies that health workers would identify cases of priority infectious diseases and report them regularly to the next level of the health system [8]. However, this innovative program is largely paper-based which lacks timely reporting of disease surveillance data, faces serious challenges and has been reported to be generally inefficient and error-prone with low completeness [8,9]. Therefore the collection of disease data via mobile phone applications could be a significant inflection point for district and national health surveillance [8,10]. In sub-Saharan African countries, mobile networks have reached far more people than any other advanced communication technology, extending far beyond the reach of electrical grid and health infrastructure in many areas [1,11]. The International Telecommunication Union (ITU) reported that Africa was the world region with a very strong widening of the regional digital divide for the period of 2011–2012 [12]. Furthermore the Information and Communication Technology (ICT) development index ranks three sub-Saharan African countries among the most dynamic [12], and mobile penetrations in Africa are still expected to observe enormous growth [2]. Although there is a global consensus that the application of mobile phone-based mHealth approaches may ultimately drive significant advancement in disease surveillance systems, little is known about current practice in developing countries, especially in sub-Sahara Africa where mobile penetration is growing at an unprecedented rate. 

## 2. Objectives and Significance of This Study 

The objective of this study was to conduct a systematic review of literature on the use of mobile phone-based eHealth approaches (SMS-based, app-based, VRS-based, and telephony-based) which have been applied in the field of health surveillance in the region. 

Mobile Health is an up-and-coming field in Public Health Surveillance and mobile-phone-based projects are mostly in pilot phases, or have recently been implemented. Consequently the extent of these projects is too limited to allow a precise and complete assessment of their impacts. However, the lack of information does not hamper the understanding of limitations, challenges and reasons for success or failure of mHealth projects. By identifying current practice, assessing the representativeness of studies included in the review, we aim to contribute to a better understanding of factors that determine successful (or failed) mHealth implementation and adaption in sub-Saharan Africa. 

## 3. Methods

### 3.1. Exclusion- and Inclusion Criteria for Considering Studies for This Review 

Information and literature gathering took place between October 2013 and January 2014. Our research was limited to studies applied in sub-Saharan African countries and in the field of surveillance. For purpose of this review we defined “sub-Saharan Africa” according to the classification of the World Bank which includes a total of 47 countries (developing only) [13]. The field of surveillance was defined according to the definition of the World Health Organization as a continuous, systematic and periodic collection, analysis and interpretation of health related data [14]. The detailed inclusion and exclusion criteria applied to this review are illustrated in Table 1. 

**Table 1:** Inclusion and exclusion criteria for identified studies.

Inclusion
Papers on the subject of mobile phone-based surveillance systems and its implementation that meets the following criteria were included:
Peer-reviewed studies, project reports and conference reports/proceedings
Written in English
Applied in sub-Sahara Africa
Basic mobile phone-, smart phone-, or tablet-based approaches which were either SMS-based, app-based, telephony-based or based on an Interactive Voice Response (IVR) System
**Exclusion**
Technical reports and presentation of hypothetical scenarios or mHealth training workshops
Discussion of literature for the purpose of theory building or critique
Summaries of the literature for the purpose of information or commentary
Editorial discussion that discussed the field to argue for research need or a course of action
Studies that were in the field of from the agricultural sector/One Health approaches (approaches which include the human and animal sector)
Studies with a timeframe with less than 7 days
Studies only mentioned trough homepages/web pages that comprised limited project information

### 3.2. Search Strategies for Identification of Studies

Literature search strategy consisted of six steps and included electronic database search of peer-reviewed bibliographic as well as non-bibliographic databases and open search on websites of reliable institutions, email request for information made to specified organizations and personal contact to authors working in the field as well as snowballing to further publications (Table 2). In order to unify search terms and formulate a uniform search strategy, the Medical Subject Headings (MeSH) of MEDLINE was used wherever possible. Search terms were grouped into three categories of interest: eHealth/mHealth specific terms, surveillance specific and geographical terms. Each category of search terms was used as single items or in combination by using the Boolean operators “AND” and “OR”. 

**Table 2:** Systematic literature and information search strategy.

Step	Description	
1	Bibliographic databases:	(1) General bibliographic databases MEDLINE, EMBASE, and PsychInfo (2) The subject-specific bibliographic database IEE Xplore (3) The African regional bibliographic database African Index Medicus (AIM)
2	Full-text journals and other non-bibliographic databases:	BioMed Central, PubMed Central (PMC), the Public Library of Science (PLoS), as well as IRIS; the digital library of WHO’s published material and technical information in full text
3	Open search on websites:	(1) World Health Organization (WHO) (2) United Nations (UN) and related sub-organizations: United Nations Children’s Fund (UNICEF) United Nations Development Programme (UNDP) United Nations Human Settlements Programme (UN-HABITAT) United Nations World Food Programme (WFP) (3) South African Centre of Infectious Disease Surveillance (SACIDS) (4) Electronic library of the World Bank (5) Care International (6) World Vision International (7) Save the Children
4	Information request via Email to specified organizations:	(1) Correspondence to all WHO country offices (2) African Developing Bank (3) South African Centre of Infectious Disease Surveillance (SACIDS) (4) Care International (5) World Vision International
5	Personal information request via Email to authors of important studies respective full texts and/or additional information and helpful hints and expertise	
6	Check through the reference lists of important studies	

The following combinations of search terms were applied:

Telemedicine OR mHealth OR mobile Health OR electronic* Health OR eHealth OR cell phone* OR cellular phone* OR mobile phone* OR texting OR message* OR SMS OR Interactive Voice Response OR IVR OR app OR VRS AND monitor* OR surveillance* AND sub-Sahara* Africa OR Africa OR (the 47 country names of sub-Saharan Africa). 

## 4. Results

Our search identified *n* = 265 publications of which *n* = 217 were identified through data bases (search steps 1 and 2, Table 2) and *n* = 48 from open search on websites and personal contacts (search steps 3–5, Table 2). In step 1, we excluded *n* = 111 articles after revision of language, title and deletion of duplicates. After reviewing the remaining *n* = 154 publications [15,16,17,18,19,20,21,22,23,24,25,26,27,28,29,30,31,32,33,34,35,36,37,38,39,40,41,42,43,44,45,46,47,48,49,50,51,52,53,54,55,56,57,58,59,60,61,62,63,64,65,66,67,68,69,70,71,72,73,74,75,76,77,78,79,80,81,82,83,84,85,86,87,88,89,90,91,92,93,94,95,96,97,98,99,100,101,102,103,104,105,106,107,108,109,110,111,112,113,114,115,116,117,118,119,120,121,122,123,124,125,126,127,128,129,130,131,132,133,134,135,136,137,138,139,140,141,142,143,144,145,146,147,148,149,150,151,152,153,154,155,156,157,158,159,160,161,162,163,164,165,166,167,168,169] a further *n* = 145 were excluded because they did not meet the inclusion criteria listed in Table 1. In the final selection stage, where any doubt was cleared by detailed discussion among the authors, we included one additional study from the list of references in one of the publication. Ultimately, we identified nine studies which fully met our inclusion criteria and were consequently included in this review.

### 4.1. Type of Studies

Seven out of nine studies were pilot or case studies [8,79,115,170,171,172,173] with a relatively short timeframe which varied between five months [8] and fifteen months for the entire operation [171], while one pilot study still in progress [172]. Two studies were longitudinal and implemented on a wider scale [128]. All studies included in the review were published within the timeframe of 2009 to 2013. 

### 4.2. Type of Participants and Data Flow 

Studies spanned across eight sub-Saharan African countries: in Botswana [65,171], Kenya [172], Madagascar [128], Malawi [170], Rwanda [115], Tanzania [8], Uganda [173] and Zambia [79]. Data were collected and transmitted in all studies by third parties including Community Health Workers (CHWs) [65,115,171,172], nurses [79], Health Surveillance Assistants (HSAs) [170], or Sentinel General Practitioners (SGPs) [128] supporting health clinics, district- as well as sub-district hospitals. None of the studies collected data directly from the participants in the community; and in all of them; interventions health workers conducted the data collection. The identified surveillance systems followed the same standard flow of information: (1) Data were either collected during standard routine data collection at the healthcare facilities or in the community by outreach personal, (2) entered into the mobile device and forwarded to a central server located at district or national level, and (3) data were analyzed and shared with different stakeholders. In some studies, a website hosting the surveillance information was used as information tool for stakeholders at national and international level. The quantity of selected organizations for data collection varied between 12 [79] and 147 health centers [173] and each used a different number of health workers for data collection. In the studies, data collectors were trained accordingly before implementation of the program and one study created poster and training cards to serve as reference materials during operation [170]. One study conducted inception meetings and focus group discussions with stakeholders before project commencement [170]. Table 3 provides an overview of the methodological characteristics of included studies. 

**Table 3:** Methodological characteristics of included studies.

Factors	No. of Publication/Citation
**1. Focus**	
1.1. Infectious disease surveillance	
Malaria	[79,171,172,173]
Tuberculosis	[65]
Influenza like-illness	[128]
1.2. Non-infectious disease surveillance	
Child malnutrition	[170,172]
Maternal health	[115]
1.3. Other (various diseases /symptoms)	[8]
**2. Design**	
Pilot/case study	[8,79,170,171,172,173]
Longitudinal study	[115,128]
**3. Hardware format **	
Basic mobile phones	[8,79,115,128,170,173]
Smart phones	[171,172]
Tablets	[65]
**4. Software / data transformation format**	
Standard 160-character SMS	[79,128,171]
Open source software “Rapid SMS”	[115,170,172,173]
App / mobile phone module	[8,65]
Email	[171]

### 4.3. Purpose of Intervention 

All studies included in this review focused on collection and transmission of disease information on a timely basis and aimed to bridge the health communication between district-, community-, and national levels. Most of the reviewed studies focused on the surveillance of infectious diseases. Four of nine studies focused on malaria surveillance only while [79,128,171,172,173], another which focused on Tuberculosis tracing (TB) [65]. The last study in the field of infectious diseases focused on monitoring of influenza like illnesses and malaria surveillance [128]. Studies in the field of non-infectious symptoms and diseases focused on child malnutrition surveillance [170,172] as well as maternal health [115]. One study focussed on improving the Integrated Disease Surveillance and Response Strategy of the WHO by reporting weekly records of deaths and a range of diseases [8] (see Table 3). Reporting time to the central server and/or next-level of authority varied between once a day and once a week according to the studies we reviewed. In Madagascar, data were transmitted immediately (at least once a day) [128]. The malaria surveillance study in Botswana applied immediate case-based notification per confirmed positive malaria case and provided additional weekly information for aggregated data [171]. In four studies, the recording time was fixed to once a week [8,79,173]. One study which focused on monitoring maternal health did not fix the recording time; data were recorded whenever a home visit took place or in case of an emergency [115]. Other studies focused on the transmission of diseases, diagnoses and/or syndromes. Cases of diseases were defined as per WHO definition [8]. Diagnoses of malaria were done by application of the Rapid Diagnosis Test (RDT) for malaria [128,171,172]. Syndrome-based data which targeted influenza-like illness (ILI) or TB defined outcomes by a combination of defined symptoms [128]. Studies focusing on child- and maternal health provided anthropometric measurements of children [170,172] as well as specific maternal and child health events [115]. 

### 4.4. Mobile Devices and Technology Applied

The majority of reviewed studies applied standard low-cost basic mobile phones for data collection [8,79,115,128,170,173], whereas two out of eight studies applied smart phones for data collection [171,172] and one study applied tablets [65] (Table 3). The mobile phones used were either private phones of staff of the participating health centers or provided by study facilitators. While eight (8) studies applied the data consignment via short messages (SMS), only one study was app-based [65]. However, different mobile-based SMS technologies were applied. Half of surveillance systems used the free and open-source software package RapidSMS for data transmission [115,170,172,173] which can be applied flexibly with regard to the level of functionality. The other 50 percent of systems used standard 160-character text messages [79,128,171] or mobile phone module [8] of applied database to transmit data. None of the studies applied a commercial software package. Most studies collaborated with local mobile phone service providers and disease information was gathered on a central server in all studies. Information sent to the server was automatically entered into a database and checked for abnormalities in most studies. Servers were also able to automatically analyze data for requested information [170]. From the server, data were transmitted to district health offices as well as research stakeholders e.g., research institutes and/or served as a data information tool for decision making at governmental level [79,170]. A two-way communication was possible in all studies. For example instant feedback loops confirming that the data were sent or additional directions in case of maternal emergencies. Summary of monthly reports were sent back to data collectors for their information and to be transcribed to the clinic paper record in order to enable cross-checking of data [115,170,173]. GPS was used in few studies to map the location of the health facilities or the district where the health event occurred in order to allow surveillance information not only in a timely but also in a spatial manner [79,171]. Studies using the RapidSMS platform were able to do spatial mapping on the RapidSMS website [170]. 

### 4.5. Acceptance and Adherence of Implementation

General acceptance of and adherence to intervention at the community level was reported to be good, although most of the studies did not specifically evaluate the acceptance among consumers. Only one study performed routine monitoring by conducting interviews with data collectors in order to learn about the practical experiences during project implementation [170]. Another study evaluated user satisfaction among data collectors [65]. No study evaluated the acceptance among end-users. In general, health workers reported a high compliance with the use of private mobile phones for intervention [173]. Actually one study reported a general compliance of 100% among data collectors [115]. Kamanga *et al.* reported that local workers were “interested and highly cooperative” [79]. Some of the studies reported that the use of mobile phone application in the field of surveillance was a great relief [8] and empowerment [170] for the community health personal because transferring the paper-based surveillance reports via public transport was time consuming and much more expensive than the costs for transmission via mobile phones. However, it was also reported that health workers were discouraged by the new system because of challenges such as lack of electricity and/or airtime as they faced problems relating to how and who will recharge the credits for the mobile phone units [8]. The data transfer rate was reported to be dependent on the motivation of data reporting staff and facilities. Regular feedback and encouragement by the study team was also found to improve the motivation among health workers [8,79]. Introduction to the system and first evaluation were reported to be a critical part because the local health staff identified several new opportunities for a sustainable project implementation [172]. In general the reviewed studies emphasized the great importance of user motivation and technology acceptance among the users as a basic requirement for an effective program implementation [128,172]. Furthermore, three studies [18,19,22] reported a good initial acceptance of the program by stakeholders such as the Ministries [18] and mobile health companies [19]. Inter alia; in Rwanda, the results of the alert monitoring system for maternal health prompted the national Ministry of Health to develop a national scale-up plan; and at the time of study publication, the project had rolled out in 18 out of 30 districts nationwide [115].

### 4.6. Risk of Bias and Challenges 

Challenges reported in the implementation of mobile phone-based health surveillance studies in sub-Sahara Africa included a wide range of issues such as technical-, financial- and infrastructural challenges, data security as well as challenges concerning medical diagnosis and availability of disease diagnosis tools. 

For instance, studies focusing on malaria surveillance via RDT diagnosis faced challenges in RDT use rates and in stock-outs [8,79,173]. Studies reported challenges such as lapses in data collection due to medical stock outs as well as inconsistency in the proportion of malaria tests used [79]. Studies also faced technical challenges stemming from the lack of competencies in mobile-based applications on the part of the health staff [8] whereas others found a considerable level of local expertise in handling mobile phone-based applications [173]. Errors in data transmission or challenges of child registrations [172] occurred, but were reported to be quite low and the error rate was lower than in paper-based systems [170]. As the surveillance data were stored on the phone, there was also a risk of deleting the data accidentally before sending the data to the central server [8]. In some studies sensitivity and specificity of case definition were a challenge whereas other studies used validated disease diagnosis tools, such as RDT for malaria [79]. Infrastructural challenges such as instability in electricity supply and network coverage/stability [115,173] as well as telephone maintenance [115] were also observed. One study faced challenges with the open-source software which, however, were adjusted easily [115]. The lack of strong governmental commitment to innovations in general was reported to be a major disadvantage to national roll-out of the mobile phone-based health surveillance [115]. 

### 4.7. Quality and Representativeness of Evidence 

In order to assess the representativeness of the studies reviewed, we built a table to assess the methodological rigour based on attributes such as strengths and weaknesses as well as their feasibility in technical and organizational terms (Table 4). As no best practice criteria for mobile health approaches currently exist, we proposed a subjective metric to assess the quality of study independently. We focused on ten attributes in order to access methodological quality of studies included in the review. 

**Table 4:** Assessment of methodological rigour of studies.

Factors	No. of Publication/Citation								
	8	79	115	170	171	172	173	128	65
1. Objectives and criteria to measure clearly defined	x	x	x	x	x	x	x		x
2. Workshops with local team to improve the system			x	x		x	x		
3. Sustainability—inclusion of stakeholders/partnerships	x	x	x	x	x	x		x	x
4. Implementation practice (training)	x		x	x	x	x	x		
5. Feedbacks loops / two way communication	x	x	x	x	x	x	x	x	x
6. Data sent in real-time (within 24 h)					x		x	x	
7. Regular supervision to health facilities/monitoring		x	x	x	x	x			
8. Evaluation									
8.1. Evaluation of costs							x	x	x
8.2. Evaluation of satisfaction of data collectors	x			x		x			x
8.3. Evaluation of end user acceptance									
9. Pilot study design	x	x		x	x	x	x		x
10. Results based on third parties (not user- based)	x	x	x	x	x	x	x	x	x

## 5. Discussion

### 5.1. Summary of Main Results

The review brings together evidence for the collection of health surveillance data by mobile phone-based eHealth applications in sub-Saharan Africa. A total of nine (9) studies were included in the review. Out of this total, five (5) studies focused on infectious diseases surveillance only. Two (2) studies concentrated on non-infectious diseases and two (2) studies were not limited to infectious- or non-infectious diseases and focused on both within their surveillance system. Studies focused on malaria surveillance exclusively or besides other diseases (*n* = 5), TB surveillance (*n* = 1), as well as child nutrition surveillance (*n* = 2), syndromic-based surveillance of influenza-like illness (*n* = 1) and routine weekly surveillance of various diseases and syndromes as per the Integrated Disease Surveillance and Response Strategy of the WHO (*n* = 1). Six studies used basic mobile phones to capture data whereas two studies applied smart phones with only one applying tablets. Surveillance systems used short message services (SMS) or apps for data transmission with different mobile phone-based SMS technologies and software being applied. In all studies, information on diseases was gathered at a central server with the possibility of a two-way communication such as instant feedback loops confirming data input, directions in case of emergency or a summary of surveillance data. All studies collected data through trained staff, such as Community Health Workers (CHWs), nurses, Health Surveillance Assistants (HSAs) or Sentinel General Practitioners (SGPs). No study collected data directly from the participants in the community; data were collected in all surveillance systems by trained health workers. All studies were published between 2009 and 2013, alluding to an increasing interest in mobile phone-based eHealth approaches in the last years.

None of the included studies met all factors for promoting good quality (Table 4). However, all studies provided a two-way communication which has been identified as a major point of strength in program implementation in previous studies. Moreover, most of the studies included stakeholders and/or local community members in the implementation process of the program. Implementation practice and regular supervision, which were known to be important factors in mHealth implementation, were applied in most of the studies. 

However, a major weakness was that seven (6) out of nine (9) studies were pilot- or case studies and served as feasibility testing of the approaches only. Besides, the naturally limited study designs of pilot studies, the methodological quality of the studies were generally found to be weak in the majority of the studies. In some other studies, the objectives were poorly defined. For instance, whereas all of the studies in the review aimed to assess the feasibility of using mobile phone-based approaches in the field of surveillance, only very few studies explicitly explained which criteria measured feasibility. Due to the short time frame of most studies, it was difficult to gauge long-term effects of the implementations. Furthermore, all approaches were based on information gathered through third parties, thus introducing a high risk of potential bias. In ICT research, it is well known that user perceptions and adherence constitute the key barriers and indictors for successful development and implementation of eHealth approaches [1]. Consequently, user attitude, acceptance and comprehensibility become particularly important when considering the use of mobile health in the highly sensitive setting of healthcare [174]. Moreover, only very limited information of sample characteristics such as demographic information (*i.e.*, sex, age, educational status) or background information (such as knowledge of mobile phone technologies) were provided in studies which were used in this review although these characteristics are known to significantly influence the user acceptance. Additionally, studies focused on the effects of the applied system and a comprehensive participants’ evaluation of acceptance and adherence were not reported in most studies. While only one study explicitly addressed user adherence through focused group discussions, during routine monitoring and in-depth interviews with health personal in order to better understand the learning-curve of technology appliance [170], one other evaluated user satisfaction among data collectors [65]. Despite the consideration for evaluations, it should be conceded that the evaluations did not comprise the perspective of the participant’s community because only the adherence of trained health staff was discussed. Lastly, the application of mobile phone-based eHealth projects in the field of surveillance is likely to increase the importance of ethical considerations in order to protect the privacy of the participants. Though, regulations of ethical clearance, as well as confidentiality, data sharing and security were only discussed in a minority of the studies included in the review. 

### 5.2. Overall Completeness of Evidence and Potential Bias in the Review Process

In this review the available evidence on mobile phone-based eHealth applications in the field of surveillance applied in Sub-Saharan Africa was systematically collected and described. However, some limitation of the review should be considered. Firstly, in defining the search strategy a narrow focus was taken. The authors included only studies in which the intervention was exclusively taken through mobile- smart phones or tablets and took place in Sub-Saharan Africa. Studies using any other mobile device for data collection and transmission, such as PDAs, patient monitoring devices and mobile telemedicine devices were excluded. We included studies which applied tablets as mobile devices because many apps are developed at same time for mobile phones and tablets. These inclusion criteria restricted the body of evidence to only the studies included in this review and thus the review may have excluded some relevant and informative studies. Combined with the limited availability of good-quality evidence and the fact that most of the studies included in the review were pilot or case studies made it difficult to conclude on the extent to which our findings were representative and had general relevance. 

The authors applied strict search criteria to the review in order to identify studies addressing the use of mobile phone-based eHealth approaches in the field of surveillance in Sub-Saharan Africa published up to January 2014. However, to present an overview of the selected field of research was challenging for a number of reasons, including: (1) the search for evidence suggested that the market of eHealth in Sub-Saharan Africa was constantly increasing and a large amount of pilot studies and approaches were applied, (2) most projects seemed to be on small scale and fragmented and publication strategies of mobile phone-based eHealth interventions in the field of surveillance lacked organization, (3) the literature search resulted in a wide latitude of hints on ongoing or already completed projects but after contacting the contact persons it turned out that those projects had never been published and were thus excluded, (4) it was probable that a number of studies were archived in repositories of research institutes or donor organizations as reports, conference proceedings, books, abstracts or papers and were therefore missed by our inclusion criteria, (5) a multitude of studies were not published in index journals and could therefore not be found in our literature search and finally (6) a wide range of studies were only mentioned on homepages or specific web pages focusing on centralized mobile health projects and comprising only very limited project information. Additional strategies to overcome these shortcomings were to extend our literature search to include networking approaches to cover websites and personal contact to organizations (Table 2).

## 6. Conclusions

### 6.1. Implications for Practice

Surveillance systems with real-time and validated data are strongly needed to strengthen disease monitoring capacity in sub-Saharan Africa and the great potential of mHealth services to support global Public Health agendas have been recognized. However, our search for evidence suggests that mobile phone-based projects in the sector of health surveillance continue to be on small scale and fragmented. Therefore, in order to allow a wide-scale implementation of mHealth services, some basic requirements should be considered and met. First, before adopting any ICT technology, it should carefully be ensured that the implementation of services is driven by the needs of national health sectors rather than by the intense technology market pressure. Second, it should be borne in mind that ICT technologies are based and dependent on technology infrastructure which is inadequately available or weak in many sub-Saharan African countries. Partnerships between policy makers throughout the study design and implementation process as well as a reliable flow of information between stakeholders are crucial for a successful implementation, program sustainability and national scalability. Third, countries should urgently increase and improve the limited awareness of mHealth in the society as well as launch a wide-scale monitoring and surveillance services. 

### 6.2. Implications for Research

Pilot studies have been developed and implemented in various forms and in specific sub-specialties of public health, but the stock of information was found to be fragmented and not well documented. For this reason, there is a strong need to share information of lessons learnt and challenges faced, in order to allow further research to benefit from best practice and available technology documentation. We highly encourage researchers as well as international stakeholders to make their project findings available to the international community and to allow further research to be built on previous achievements. The focus should be on creating a learning environment to help build a more evidence-based field of mobile Health practice in sub-Saharan Africa. Furthermore the field of user acceptance, adherence and comprehensibility is presently neglected and should be prioritized in future research. Due to the high importance of implementing a new ICT in the health sector, this process requires further attention in future research. Benefits of mobile phone-based eHealth applications such as its efficiency, low delivery costs as well as timeliness of service delivery and data management also need to be further explored. As the evidence found in our review shows that the studies were of low methodological quality, we generally favour a strong demand for more high quality research such as e.g., randomized trials in the field of mobile phone-based surveillance. 
